# Commentary: Aiming for Study Comparability in Parkinson's Disease: Proposal for a Modular Set of Biomarker Assessments to be Used in Longitudinal Studies

**DOI:** 10.3389/fnagi.2016.00331

**Published:** 2017-01-09

**Authors:** Yassar Alamri, Michael MacAskill, Tim Anderson

**Affiliations:** Department of Medicine, University of OtagoChristchurch, New Zealand

**Keywords:** dementia, Parkinson disease, cohort studies, markers, harmonization

## Introduction

We read the conclusions of Lerche et al. (2016) with great interest. Various definitions exist in the literature for biological markers (often shortened to biomarkers). In medicine, a biomarker may refer to an indicator based upon which an inference about the person's health can be made. The National Health Institute's Biomarkers Definitions Working Group (Biomarkers Definitions Working Group, 2001) defines a biomarker as “a characteristic that is objectively measured and evaluated as an indicator of normal biological processes, pathogenic processes or pharmacological responses to a therapeutic intervention.”

**Figure 1:**
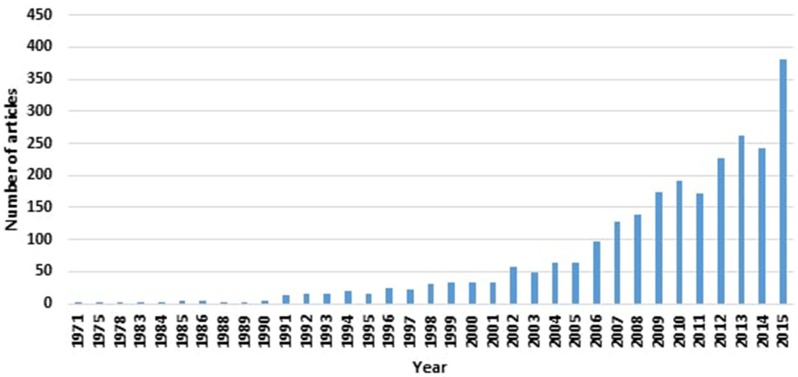
**Increasing numbers of PD biomarker-related articles, as indexed by MEDLINE® as of January 2016, are appearing in the literature**.

Despite the recent interest in finding biomarkers for Parkinson's disease (PD; as evident from the number of “Parkinson's disease biomarker” articles indexed by the MEDLINE® Database; see Figure 1), an acceptable biomarker for PD remains to be elusive. Given the complexity underpinning PD's pathological processes, a useful single biomarker is unlikely to encompass the plethora of disease facets. However, the diverse features of PD's aetiological process and manifestations lend it particularly suited to have a number of biomarkers (of different categories and types) that serve distinct purposes at various stages of the disease.

## Challenges facing PD biomarker research

As with other neurodegenerative disorders, several hurdles face biomarker discovery in PD. Such challenges include the general complexity of the human CNS, limited access and availability of tissue for histological diagnosis during the patient's lifetime and the restricted number of clinical end-points and the lack of validation models for present ones (Dunckley et al., 2005). Added to these general obstacles are the diagnostic uncertainties surrounding PD, including disease heterogeneity and the potential for atypical parkinsonian syndromes.

The multiplicity of disease aspects of PD make it difficult for a single all-purpose biomarker to ever exist. This is evident from the current lack of such a “Holy Grail” biomarker to date. By the same token, “reductionist”-type biomarkers focusing on single aspects of PD molecular neuropathology are unlikely to be overly clinically useful in the general schema of disease (Mielke and Maetzler, 2014). It seems, therefore, that several biomarkers of different types may be utilized in conjunction to identify the person's disease stage.

Taking PD diagnosis as an example, no one single biomarker to date has demonstrated perfect sensitivity and specificity; even post-mortem pathological examinations can be inconclusive at times (Lees et al., 2009; Berg et al., 2013). Instead, the suggested tier-based system of different types of biomarkers (e.g., clinical assessment by a specialist, biofluid analysis, genetic testing and/or imaging studies) used in concert is much more likely to yield much needed diagnostic accuracy (Streffer et al., 2012; Schlossmacher and Mollenhauer, 2014).

## Future directions

Much research has gone into obtaining new prospectively-collected data (e.g., The Parkinson Progression Marker Initiative Marek, 2011), as well as examining archived biofluid and tissue specimens [e.g., Honolulu Asian Aging Study (Abbott et al., 2016)]. However, much still remains to be desired.

The ongoing collaborative efforts are hoped to generate large datasets and standardized resources which are publicly available. The next major step is the proper utilization of such data mines. This would involve using current bioinformatics and technological advances (i.e., -omics) to thoroughly evaluate the data, as well as making use of integration models which can capture interplay between biomarkers that would have otherwise been hidden (Azuaje, 2011).

## Author contributions

Study concept and design: YA, TA, and MM; Acquisition of data: YA; Analysis and interpretation: YA, MM; Critical revision of manuscript: YA, TA, and MM; Study supervision: TA, MM.

### Conflict of interest statement

The authors declare that the research was conducted in the absence of any commercial or financial relationships that could be construed as a potential conflict of interest. The reviewer ND and handling Editor declared their shared affiliation, and the handling Editor states that the process nevertheless met the standards of a fair and objective review.
